# Comparison of Sutureless and Sutured Aortic Valve Replacements in Patients with Redo Infective Endocarditis

**DOI:** 10.3390/medicina60122037

**Published:** 2024-12-11

**Authors:** Cagdas Baran, Ahmet Kayan, Canan Soykan Baran, Ali Fuat Karacuha, Sadik Eryilmaz

**Affiliations:** 1 Department of Cardiovascular Surgery, Heart Center, Cebeci Hospitals, Ankara University School of Medicine, 06230 Ankara, Turkey; cagdasbaran@gmail.com (C.B.); sadikeryilmaz@gmail.com (S.E.); 2 Department of Cardiovascular Surgery, Kirikkale High Specialization Hospital, 71300 Kirikkale, Turkey; 3 Department of Cardiovascular Surgery, Ankara 29 Mayıs Hospital, 06105 Ankara, Turkey; canansykn@hotmail.com; 4 Department of Cardiovascular Surgery, Trabzon Kanuni Education and Research Hospital, 61250 Trabzon, Turkey; alifuatkaracuha@hotmail.com

**Keywords:** aortic valve replacement, biological prosthesis, sutureless valve

## Abstract

*Background and Objectives*: This study aims to assess the postoperative outcomes and complications of sutureless and sutured aortic valve replacement in patients with infective endocarditis. *Materials and Methods*: A total of 58 patients who underwent redo aortic valve replacement for bacterial or non-bacterial endocarditis between January 2018 and March 2023 were included in our study. Surgical procedures were performed through a full median sternotomy due to redo cases and to provide optimal access. Demographic characteristics, operative times, postoperative complications and some echocardiographic data were compared. All cases were meticulously evaluated preoperatively by a cardiac team to select the best treatment option. *Results*: The mean ICU length of stay was significantly shorter in the sutureless valve group at 5.4 ± 3.9 days compared to 7.9 ± 4.1 days in the sutured valve group (*p* = 0.029). However, the sutureless group had a mean operation time of 164.7 ± 37.3 min, while the sutured group had a mean operation time of 197.7 ± 45.6 min (*p* = 0.044). Again, the difference in cardiopulmonary bypass times between the two groups was statistically significant (*p* = 0.039). And again, four (14.2%) patients in the sutureless group underwent reoperation due to bleeding, while eight (26.6%) patients in the sutured group underwent postoperative bleeding control (*p* = 0.048). *Conclusions*: Our study suggests that sutureless aortic valve replacement may offer advantages in terms of operative efficiency and postoperative recovery compared to conventional sutured valves, with some significant differences in terms of some complications.

## 1. Introduction

Infective endocarditis is a serious condition characterized by inflammation of the inner lining of the heart, often leading to severe complications and notably high mortality rates, particularly in patients who have previously experienced embolic events [1]. The emergence of antibiotic resistance, combined with advancements in surgical techniques, has played a critical role in contributing to a gradual decline in mortality rates associated with this condition in recent years [2]. However, in cases of advanced infective endocarditis, the treatment necessitates not only robust antibiotic therapy but also surgical intervention. This typically involves the removal of the infected valve, followed by its replacement to restore proper cardiac function [3].

Patients presenting with active infective endocarditis often endure prolonged periods of cardiopulmonary bypass and aortic cross-clamping during surgical procedures. These factors, coupled with the potential for organ damage stemming from sepsis, contribute to elevated morbidity and mortality rates [4]. Moreover, the introduction of sutureless aortic valves has significantly enhanced the ability to address cases involving small aortic annuli, allowing for more straightforward implementation of minimally invasive approaches [5]. This innovation is particularly beneficial, as it reduces the surgical trauma associated with traditional techniques.

Recent developments in medical technology have profoundly transformed the management of infective endocarditis. For example, the utilization of sutureless aortic valves has revolutionized surgical practices, enabling more effective treatment of complex cases. This advancement not only streamlines the surgical process but also improves patient outcomes by decreasing the necessity for extensive incisions and facilitating quicker recovery times. Consequently, patients are now able to benefit from less invasive techniques while still receiving comprehensive care tailored to their condition.

Despite these promising advancements in antibiotic and surgical therapies, infective endocarditis remains a condition fraught with significant challenges. The complexity of the disease and the high risk of complications highlight the urgent need for ongoing research and innovation in both medical and surgical interventions. By continuing to explore new strategies and technologies, we can enhance treatment efficacy and improve patient prognosis in the face of this formidable disease. In this study, we aimed to compare the results and complications of sutureless and sutured aortic valve replacement in patients with infective endocarditis.

## 2. Materials and Methods

Between January 2018 and March 2023, a total of 58 patients underwent redo aortic valve replacement for bacterial or non-bacterial endocarditis at our institution. The Perceval aortic valve prosthesis (LivaNova, Saluggia, Italy) was used in all patients with a sutureless valve. One patient had a previous transcatheter aortic valve implantation (TAVI) procedure. Two patients had previously undergone Perceval valve implantation. Perceval valve reoperation was performed in 28 patients who developed endocarditis. In the remaining 30 patients, bioprosthetic or mechanically sutured valves were used depending on their condition. In our study of 58 patients, 20 (34.5%) had non-bacterial endocarditis, while 38 (65.5%) had bacterial endocarditis. Among the bacterial cases, Staphylococcus aureus was the most common pathogen, affecting 17 patients, followed by Streptococcus species in 10 patients, Enterococci in 5 patients and coagulase-negative staphylococci (KNS) in 3 patients. Additionally, 3 patients had endocarditis caused by other less common pathogens. Identifying the specific causative bacteria is essential for tailoring antibiotic therapy and determining the appropriate surgical approach. All cases were evaluated by a cardiac team consisting of a cardiac surgeon, cardiologist, anesthesiologist and infectious disease specialist. Written informed consent was obtained from all patients. This single-center retrospective study was approved by the institutional ethics committee and adhered to the principles outlined in the Declaration of Helsinki (Human research ethics committee details; approval date, protocol number: 13 September 2024, 2024/564).

### 2.1. Patients

The patients included in this study were those diagnosed with active infective endocarditis, either bacterial or non-bacterial, who required redo aortic valve replacement due to infective endocarditis after previously undergoing cardiac surgery. The diagnosis of infective endocarditis was established based on clinical symptoms, microbiological cultures, echocardiographic findings and major and minor criteria, according to the modified Duke criteria. All participants were aged 18 years or older. Patient eligibility was assessed by a multidisciplinary team consisting of a cardiac surgeon, cardiologist, anesthesiologist and infectious disease specialist. Only patients deemed suitable for surgery based on their overall health, comorbidities and the severity of infective endocarditis were included. Written informed consent was obtained from all patients or their legal representatives, following approval from the institutional ethics committee.

Patients with severe and uncontrolled comorbidities, such as advanced renal failure, uncontrolled diabetes or end-stage heart failure, which could significantly increase surgical risks or interfere with postoperative recovery, were excluded from the study. Patients with active malignancies were also excluded, as cancer treatments or metastasis could complicate surgical outcomes and recovery. Those with contraindications for cardiac surgery, including uncorrectable coronary artery disease, severe peripheral vascular disease or significant aortic root dilatation that made surgery technically unfeasible, were not included in the study. Pregnant or lactating women were excluded due to the potential risks associated with surgery and anesthesia. Patients with known severe allergic reactions to prosthetic valve materials or surgical components were excluded as well. Lastly, patients who were unlikely to follow up or who were lost to follow-up after surgery were excluded from the study.

The selection between Perceval sutureless valves and traditional prosthetic valves is guided by factors such as annular size and calcification, the need for redo surgery, infection control and the specific anatomical features of the patient. Both valve types have their place in clinical practice, with Perceval offering advantages in terms of shorter surgical times and less tissue trauma, particularly in patients with small or irregular annuli. However, in cases with significant calcification or complex anatomical features, traditional sutured valves remain the preferred choice. In patients with infective endocarditis, careful patient selection and proper infection control are critical when considering the use of Perceval sutureless valves.

### 2.2. Surgical Technique

The surgical procedure started with a sternotomy because it provides an optimal view of the heart and because it was the second operation of the patients. After general anesthesia, the patient was placed in the supine position on the operating table. After a longitudinal incision was made in the middle of the sternum, the sternum was cut with a sternal saw. The pericardium was opened, and the heart was exposed. After adequate visibility was achieved, cardiopulmonary bypass (CPB) was initiated. The ascending aorta and right atrium were cannulated. Aortic cross-clamp was applied to isolate the aorta, and cardioplegia was administered to induce cardiac arrest and protect the myocardium throughout the procedure.

**Figure 1:**
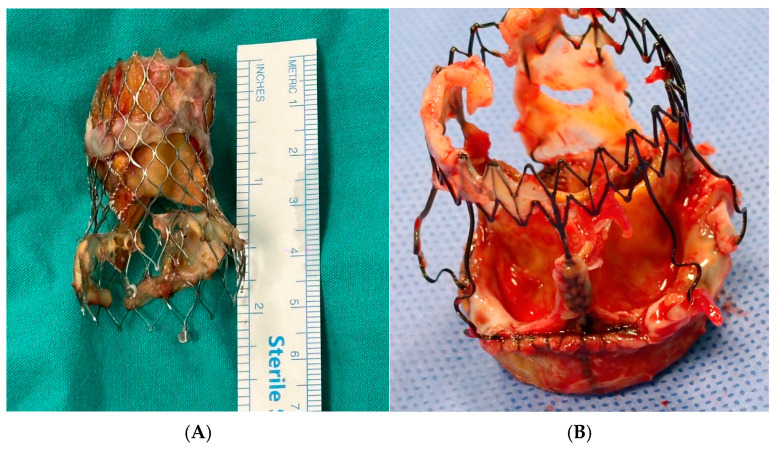
(**A**) Infective endocarditis after transcatheter aortic valve endocarditis: This image illustrates the surgical process of removing a previously implanted transcatheter aortic valve. (**B**) Infective endocarditis after Perceval valve endocarditis: This image depicts the Perceval valve after its removal from the patient.

After the heart was still and bleeding-free, the aortic valve was carefully examined, and the native valve was assessed for calcification or other pathologic features. The annulus was prepared via removal of the infected valve and removal of calcified material. Figure 1 shows the valves from patients with infective endocarditis after TAVI and after Perceval.

In 28 patients, a sutureless Perceval valve was placed in the aortic annulus with minimal use of sutures. The design of the valve allowed for rapid deployment, and optimal alignment and fit were achieved. The valve was appropriately positioned in the annulus with natural expansion of the stent frame.

In 30 patients, a traditional sutured prosthetic valve was used. In these cases, the surgical technique involved more extensive suturing procedures, which is a standard approach for traditional valve replacements. Sutured valves were selected based on anatomical measurements of the patients and implanted with established techniques to ensure effective functionality.

In both groups, the aortic cross-clamp was removed after valve implantation, and the heart was revived with a gradual return of blood flow. The quality of valve function and hemodynamic performance were assessed both visually and via transesophageal echocardiography (TEE). The pericardium was closed, and the sternum was reapproximated with wire sutures. After the end of the procedure, CPB was gradually withdrawn. The patient was transferred to the intensive care unit (ICU) for close postoperative follow-up.

Our study included the use of both sutureless Perceval valves and conventional sutured prosthetic valves through sternotomy for aortic valve replacement. The choice of sternotomy provided optimal conditions for these complex procedures, as all patients in the study had previous open-heart surgery.

### 2.3. Statistical Analysis

Demographic information and patient characteristics were analyzed using IBM SPSS version 15.0. The normality of the data was assessed using the Shapiro–Wilk test. Quantitative data were reported based on their distribution, presented either as mean and standard deviation (SD) or as median and interquartile range (IQR), depending on the normality of the data. For categorical variables, the results are expressed as frequencies and percentages. Comparisons between groups for categorical data were conducted using the chi-square test, while ANOVA was employed for comparing means across multiple groups.

## 3. Results

The clinical and demographic characteristics of the patients included in our study are presented in Table 1. A total of 11 patients (39.2%) in the unsutured valve group were female, and 14 patients (46.6%) in the sutured group were female (*p* = 0.353). The mean age of participants in the seamless valve group was 72.12 ± 8.34 years, while the mean age in the stitched valve group was 68.22 ± 7.78 years (*p* = 0.212). The comorbidities in both groups were similar. Peripheral arterial disease was present in nine patients (32.1%) in the unsewn valve group and eight patients (26.6%) in the sutured valve group (*p* = 0.456). The prevalence of diabetes was seven patients (25%) in the unsutured group and six patients (20%) in the sutured group (*p* = 0.938). Of note, pulmonary hypertension was seen in one patient (3.5%) in the unsutured group and one patient (3.3%) in the sutured group (*p* = 0.865). In addition, arterial hypertension was reported in nine (32.1%) unsutured group patients and eight (26.6%) sutured group patients (*p* = 0.634).

In terms of the ejection fraction and New York Heart Association (NYHA) classification, both groups showed statistically significant and comparable results (Table 1). The mean maximum aortic valve gradient was 66.7 ± 24.5 mmHg for the unsutured group and 64.9 ± 27.6 mmHg for the sutured group (*p* = 0.554). The mean aortic valve gradient was 38.6 ± 17.6 mmHg for the unsutured group and 39.5 ± 14.7 mmHg for the sutured group (*p* = 0.801). When we compared the WBC, CRP and procalcitonin values of the patients, no statistical difference was found between both groups (Table 1).

The mean size of the vegetations was 0.8 ± 0.9 cm in the unsutured group and 1.2 ± 0.8 cm in the sutured group (*p* = 0.088). Atrial fibrillation was found in nine patients (32.1%) in the unsutured group and eight patients (26.6%) in the sutured group (*p* = 0.604). Sinus rhythm was preserved in 19 patients (67.8%) in the unsutured group and 22 patients (73.3%) in the sutured group, with no significant difference (*p* = 0.492).

As one of the most notable results in our study, the duration of surgery was significantly shorter in the sutureless valve group; the mean duration was 164.7 ± 37.3 min for the sutureless group and 197.7 ± 45.6 min for the sutured group (*p* = 0.044). In parallel with this result, the duration of cardiopulmonary bypass (CPB) was also significantly shorter in the sutureless group; the mean duration was 75.6 ± 29.4 min for the sutureless group and 104.4 ± 35.4 min for the sutured group (*p* = 0.039) (Table 2).

The reoperation rate due to bleeding was four patients (14.2%) in the unsutured valve group and eight patients (26.6%) in the sutured valve group (*p* = 0.048). In terms of stroke incidence, one patient (3.5%) had a stroke in the unsutured group compared to three patients (10%) in the sutured group (*p* = 0.502). Acute renal failure was reported in four patients (14.2%) in the unsutured group and seven patients (23.3%) in the sutured group (*p* = 0.433) (Table 3).

**Figure 2:**
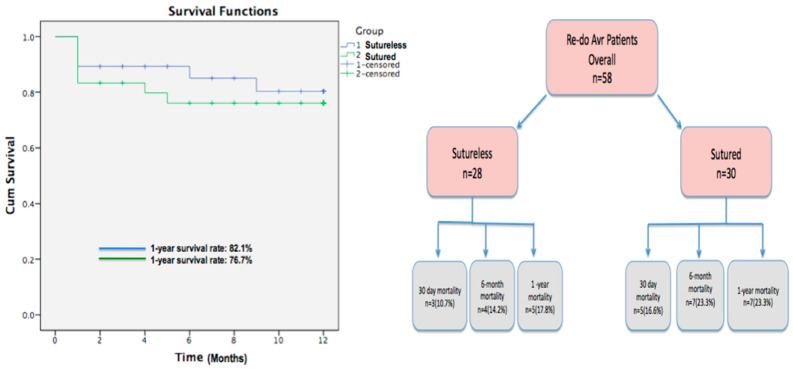
Mortality rates during follow-up of patients.

The mean duration of intensive care unit (ICU) stay was 5.4 ± 3.9 days in the sutureless valve group and 7.9 ± 4.1 days in the sutured group (*p* = 0.029), indicating that patients who underwent the sutureless procedure had a better recovery time than patients in the other group. The 30-day mortality rates were three patients (10.7%) in the sutureless group and five patients (16.6%) in the sutured group (*p* = 0.089). At the 6-month follow-up, the mortality rates were four patients (14.2%) in the unsutured group and seven patients (23.3%) in the sutured group (*p* = 0.073) (Figure 2).

In conclusion, the sutureless valve group shows some advantages in terms of reoperation rates and the length of intensive care unit stay, but more comprehensive and multicenter studies should be performed in the light of this study for clearer and more reliable results.

## 4. Discussion

In our study, we compared the use of a sutureless Perceval valve in 28 patients with a sutured prosthetic valve and in 30 patients with redo aortic valve replacement. Our findings demonstrated that the sutureless approach offers multiple advantages in terms of operational efficiency and patient recovery.

Historically, the use of sutureless valves, including the Perceval valve, was contraindicated in patients with infective endocarditis due to concerns about valve fixation and the potential for residual infection at the surgical site. These concerns were primarily related to the challenge of securing the valve in the presence of ongoing infection, which could predispose the patient to paravalvular leaks or even recurrent infection. However, recent evidence and clinical experience have led to a reconsideration of these initial reservations. As newer techniques for valve implantation have improved, and as our understanding of the perioperative management of endocarditis patients has evolved, the use of sutureless valves like Perceval has become more acceptable in select cases [6]. In particular, Perceval sutureless valves have demonstrated several advantages in patients undergoing redo valve replacement surgery, including those with infective endocarditis. These advantages include faster implantation, reduced operation time and the potential for less trauma to the surrounding tissues compared with traditional sutured valves [6]. Moreover, the Perceval valve’s self-expanding design facilitates better adaptation to the annulus, which can be particularly useful in cases of structural damage due to infection, such as in small or irregular annuli. These factors may help reduce complications and improve recovery times, which are critical in this patient group.

The shorter surgical time observed in the sutureless valve group (*p* = 0.044) is consistent with previous studies emphasizing the efficacy of sutureless techniques. This reduction in surgical time is important, as it has the potential to reduce anesthesia exposure and lower the risk of complications associated with prolonged surgeries. Similar to this result, the shorter cardiopulmonary bypass (CPB) time in the sutureless group (*p* = 0.039) supports the notion that sutureless valves provide a more robust surgical process. This study confirms that the sutureless aortic valve achieved significantly lower values in CPB and operative times, in accordance with previous literature [7,8].

The lower rate of reoperation for bleeding in the sutureless group (*p* = 0.048) suggests that this approach may reduce one of the most common and problematic complications in cardiac surgery. These findings improve patient safety and reduce the need for subsequent intervention.

In a previous study by Colarossi et al., it was shown that the pacemaker implantation rate was higher in Perceval valves, but in our study, one patient in each group needed a postoperative pacemaker [9]. Statistically, there was no difference between the two groups (*p* = 0.798). Of course, these previous studies were performed in patients who underwent relatively less risky first surgery, and our number of patients was small.

The high rate of reoperation due to bleeding in our study (12 patients in total) indicates what has been emphasized in previous studies, namely that bleeding is an important complication in complex cardiac surgery. For example, a review by Hermanns et al. found that reoperation for bleeding was a common complication, especially in patients with severe infective endocarditis, and it was associated with increased mortality and morbidity [10]. However, when we looked at both patient groups, four (14.2%) of the patients with sutureless valves and eight (26.6%) of the patients with sutured valves were reoperated for bleeding (*p* = 0.048). This is due to the fact that sutureless valves allow this complication to occur at a lower rate.

The negative prognostic impact of embolic events and heart failure in patients with infective endocarditis undergoing surgery has been well documented in previous studies. Pizzino et al. highlighted that embolic events, such as stroke or peripheral embolism, are significant predictors of poor outcomes in patients with infective endocarditis, leading to increased mortality rates and longer recovery times after surgery [11]. In their study, embolic events were found to substantially increase the complexity of surgical interventions, contributing to higher rates of postoperative complications, including neurological deficits and prolonged intensive care unit (ICU) stays.

Similarly, heart failure is another critical factor that negatively impacts the prognosis of these patients. Heart failure often exacerbates the difficulty of surgery, particularly in valve replacement procedures, by reducing the heart’s ability to tolerate the hemodynamic changes during and after the operation. The presence of heart failure in infective endocarditis patients further complicates the postoperative recovery, increasing the likelihood of adverse outcomes, such as prolonged mechanical ventilation, renal failure and higher mortality rates [11].

In our study, although we did not specifically assess the prognostic role of embolic events and heart failure in the context of valve replacement, our findings align with previous reports suggesting that these factors should be closely monitored in surgical decision making. Patients presenting with these comorbidities may require more intensive perioperative management and careful postoperative monitoring to optimize outcomes.

Stroke was observed in one (3.5%) patient in the unsutured group and three (10%) patients in the sutured group (*p* = 0.502). Stroke rates after cardiac surgery vary, but a study by Asta L et al. involving 580,117 patients showed that the incidence of cerebrovascular events was 1.85%. Furthermore, recent findings emphasize that a multidisciplinary approach in the perioperative period can further reduce the incidence of stroke, highlighting the need for coordinated care in high-risk surgeries [12]. Paravalvular leaks were mild in only one patient, suggesting that surgical techniques and valve choices are effective in preventing this complication. According to a study by Saito et al., paravalvular leak can significantly affect patient outcomes, and prevention of these leaks is critical to ensuring long-term valve function [13]. In our study, acute renal failure developed in four (14.2%) patients in the unsutured group and seven (23.3%) patients in the sutured group, which is a worrying finding (*p* = 0.433). Previous studies by Hu et al. showed that acute kidney injury is a common complication, especially in patients undergoing major cardiac surgery [14].

The choice of valve in patients undergoing aortic valve replacement, particularly in those with a history of infective endocarditis, plays a critical role in determining the risk of postoperative thrombosis. While sutureless Perceval valves have shown significant benefits in terms of surgical time and recovery, it is important to consider their potential thrombotic risks. In patients with mechanical valves, thrombosis is a well-recognized complication, often leading to valve dysfunction or thromboembolic events, such as stroke.

One of the key tools in assessing the risk of thrombosis in patients with mechanical heart valves is the CHA2DS2-VASc score, a clinical risk stratification system traditionally used to evaluate stroke risk in patients with atrial fibrillation. However, the CHA2DS2-VASc score has also been shown to be predictive of thrombotic complications in patients with mechanical heart valves. According to a study titled “The Predictive Value of the CHA2DS2-VASc Score in Patients with Mechanical Mitral Valve Thrombosis” by Çınar et al., the CHA2DS2-VASc score was found to correlate with the risk of developing thrombotic events in patients with mechanical valves [15]. The study demonstrated that a higher CHA2DS2-VASc score was associated with a significantly increased risk of thrombotic complications, including thrombosis of mechanical mitral valves.

While the Perceval valve is a bioprosthetic valve and typically does not carry the same thrombosis risk as mechanical valves, patients who undergo surgery with any type of valve replacement should still be carefully monitored for thromboembolic events, particularly those with high-risk scores. The presence of comorbidities, such as diabetes, hypertension and atrial fibrillation, which are part of the CHA2DS2-VASc scoring system, can elevate the risk of thrombosis postoperatively. It is crucial to apply anticoagulation therapy judiciously based on individual patient risk factors to mitigate this risk.

Although sutureless bioprosthetic valves like the Perceval valve are associated with a lower incidence of thrombosis compared to mechanical valves, clinicians must continue to consider the CHA2DS2-VASc score and other relevant clinical factors when managing anticoagulation and thrombosis prevention in patients undergoing valve replacement surgery.

In our study, the 30-day mortality was found to be three (10.7%) patients in the unsutured group and five (16.6%) patients in the sutured group (*p* = 0.089). In addition, the 6-month mortality rates were four (14.2%) patients in the unsutured group and seven (23.2%) patients in the sutured group (*p* = 0.073). These findings are consistent with the high mortality rates reported in high-risk cardiac surgeries. According to a review by Agraval et al., the mortality rates in high-risk cardiac surgery patients may be significantly affected by factors such as comorbidities and procedure complexity [16]. Of course, larger scale studies with longer follow-up are needed for a more robust comparison of mortality rates.

Our study argues that Perceval valves potentially reduce both surgical time and the associated morbidity and mortality. In particular, the use of Perceval valves designed for faster deployment in our patient groups with both redo and infective endocarditis contributes to shorter surgical times and improved recovery outcomes. Furthermore, data from comparative studies show that patients who receive a sutureless valve tend to experience fewer postoperative complications, confirming the benefits of this innovative approach [6,17]. By reducing valve placement time and minimizing the invasiveness of the procedure, the Perceval valve may have played an important role in reducing adverse outcomes specific to both infective endocarditis and reoperation.

### Study Limitations

Despite the valuable findings of this study, there are some limitations. Firstly, the small sample size of 58 patients may limit the generalizability of the results. A larger cohort may provide a better examination of the complications and outcomes between the two groups. In addition, the study was conducted in a single center and retrospectively, and multicenter and prospective studies would be more useful for validating the results and evaluating variability.

## 5. Conclusions

In conclusion, we suggest that by reducing valve placement time and minimizing the invasiveness of the procedure, the Perceval valve may have played an important role in reducing adverse outcomes specific to both infective endocarditis and reoperation. The observed morbidity and mortality rates suggest that surgical techniques and postoperative management strategies should be continuously improved. The favorable effect of the Perceval valve in reducing surgical time and improving outcomes suggests that its use in high-risk cardiac procedures may be beneficial. Future research should focus on optimizing perioperative care, exploring less invasive surgical methods and reducing the risk of complications.

## Figures and Tables

**Table 1:** Baseline patient characteristics.

Variables	Sutureless Valve (*n* = 28)	Sutured Valve (*n* = 30)	*p*
Female	11 (39.2%)	14 (46.6%)	0.353
Age, years	72.12 ± 8.34	68.22 ± 7.78	0.212
BMI, kg/m^2^	24.9 ± 4.9	23.7 ± 4.2	0.930
Peripheral arterial disease	9 (32.1%)	8 (26.6%)	0.456
Diabetes	7 (25%)	6 (20%)	0.938
Pulmonary hypertension	1 (3.5%)	1 (3.3%)	0.865
Arterial hypertension	9 (32.1%)	8 (26.6%)	0.634
COPD	6 (21.4%)	6 (20%)	0.726
History of PCI	4 (14.2%)	5 (16.6%)	0.609
Recent myocardial infarct	8 (28.5%)	10 (33.3%)	0.282
Kidney failure	5 (17.8%)	7 (23.3%)	0.339
Dialysis	1 (3.5%)	2 (6.6%)	0.988
Creatinine, mg/dL	1.7 ± 0.88	1.6 ± 0.92	0.866
WBC, 1/nL	10.5 ± 4.8	10.9 ± 5.1	0.602
CRP, mg/L	86.4 ± 70.3	87.0 ± 77.8	0.87
Procalcitonin, ng/mL	2.6 ± 6.9	3.5 ± 7.9	0.134
Ejection fraction			
Normal (>49%)	18 (64.2%)	16 (53.3%)	0.734
Moderate (31–49%)	7 (25%)	10 (33.3%)	0.182
Poor (<30%)	3 (10.7%)	4 (13.3%)	0.277
EuroSCORE II	27.6 ± 19.92	26.6 ± 18.66	0.679
NYHA functional classification			0.566
NYHA I	8 (28.5%)	10 (33.3%)	
NYHA II	9 (32.1%)	7 (23.3%)	
NYHA III	8 (28.5%)	8 (26.6%)	
NYHA IV	3 (10.7)	5 (16.6%)	
Paravalvular leakage	7 (25%)	4 (13.3%)	0.829
Aortic valve peak gradient			0.353
Mild (≤50 mmHg)	8 (28.5%)	8 (26.6%)	
Moderate (≤75 mmHg)	9 (32.1%)	9 (30%)	
Severe (>75 mmHg)	11 (39.2%)	13 (43.3%)	
Aortic valve max gradient mmHg (Mean)	66.7 ± 24.5	64.9 ± 27.6	0.554
Aortic valve mean gradient mmHg (Mean)	38.6 ± 17.6	39.5 ± 14.7	0.801

Data are presented as *n* (%) or mean ± standard deviation. BMI, body mass index; COPD, chronic obstructive pulmonary disease; PCI, percutaneous coronary intervention; NYHA, New York Heart Association class; WBC, white blood cell; CRP, C-reactive protein.

**Table 2:** Perioperative characteristics.

Variables	Sutureless Valve (*n* = 28)	Sutured Valve (*n* = 30)	*p*
Vegetation	8 (28.5%)	12 (40%)	0.122
Vegetation, cm	0.8 ± 0.9	1.2 ± 0.8	0.088
Prothesis endocarditis	13 (46.4%)	15 (50%)	0.203
Aortic regurgitation	18 (64.2%)	21 (70%)	0.199
Atrial fibrillation	9 (32.1%)	8 (26.6%)	0.604
Sinus rhythm	19 (67.8%)	22 (73.3%)	0.492
Operating time, min	164.7 ± 37.3	197.7 ± 45.6	0.044
CPB time, min	75.6 ± 29.4	104.4 ± 35.4	0.039
Concomitant procedure			
Mitral valve repair/replacement	5 (17.8%)	3 (10%)	0.731
Tricuspid valve repair	3 (10.7%)	6 (20%)	0.610
CABG	3 (10.7%)	4 (13.3%)	0.344

Data are presented as *n* (%) or mean ± standard deviation. CPB, cardiopulmonary bypass; CABG, coronary artery bypass graft surgery.

**Table 3:** Postoperative outcomes.

Variables	Sutureless Valve (*n* = 28)	Sutured Valve (*n* = 30)	*p*
Reoperation for bleeding	4 (14.2%)	8 (26.6%)	0.048
Stroke	1 (3.5%)	3 (10%)	0.502
Paravalvular leakage			
Mild	1	0	
Moderate	-	-	
Severe	-	-	
Pacemaker implantation	1 (3.5%)	1 (3.3%)	0.798
Acute kidney failure	4 (14.2%)	7 (23.3%)	0.433
Septic shock	1 (3.5%)	2 (6.6%)	0.212
Mean gradient, mmHg	7.8 ± 4.1	8.5 ± 3.8	0.909
ICU stay, days	5.4 ± 3.9	7.9 ± 4.1	0.029
30-day mortality	3 (10.7%)	5 (16.6%)	0.089
6-month mortality	4 (14.2%)	7 (23.3%)	0.073
1-year mortality	5 (17.8%)	7 (23.3%)	0.088

Data are presented as *n* (%) or mean ± standard deviation. ICU, intensive care unit.

## Data Availability

Data are contained within the article.

## References

[1] Misfeld M., Girrbach F., Etz C.D., Binner C., Aspern K.V., Dohmen P.M., Davierwala P., Pfannmueller B., Borger M.A., Mohr F.-W. (2014). Surgery for infective endocarditis complicated by cerebral embolism: A consecutive series of 375 patients. J. Thorac. Cardiovasc. Surg..

[2] Thuny F., Grisoli D., Collart F., Habib G., Raoult D. (2012). Management of infective endocarditis:Challenges and perspectives. Lancet.

[3] Habib G., Lancellotti P., Antunes M.J., Bongiorni M.G., Casalta J.P., Del Zotti F., Dulgheru R., El Khoury G., Erba P.A., Iung B. (2015). 2015 ESC Guidelines for the management of infective endocarditis:The Task Force for the Management of Infective Endocarditis of the European Society of Cardiology (ESC). Endorsed by: European Association for CardioThoracic Surgery (EACTS), the European. Eur. Heart J..

[4] Holst K.A., Dearani J.A., Burkhart H.M., Connolly H.M., Warnes C.A., Li Z., Schaff H.V. (2013). Reoperative multivalve surgery in adult congenital heart disease. Ann. Thorac. Surg..

[5] Gersak B., Fischlein T., Folliguet T.A., Meuris B., Teoh K.H.T., Moten S.C., Solinas M., Miceli A., Oberwalder P.J., Rambaldini M. (2016). Sutureless, rapid deployment valves and stented bioprosthesis in aortic valve replacement: Recommendations of an International Expert Consensus Panel. Eur. J. Cardiothorac. Surg..

[6] Zubarevich A., Amanov L., Arjomandi Rad A., Beltsios E.T., Szczechowicz M., Osswald A., Ruhparwar A., Weymann A. (2023). Single-Center Real-World Experience with Sutureless Aortic Valve Prosthesis in Isolated and Combined Procedures. J. Clin. Med..

[7] Fischlein T., Meuris B., Hakim-Meibodi K., Misfeld M., Carrel T., Zembala M., Gaggianesi S., Madonna F., Laborde F., Asch F. (2016). The sutureless aortic valve at 1 year: A large multicenter cohort study. J. Thorac. Cardiovasc. Surg..

[8] Mashhour A., Zhigalov K., Szczechowicz M., Mkalaluh S., Easo J., Eichstaedt H., Borodin D., Ennker J., Weymann A. (2018). Snugger method—The Oldenburg modification of perceval implantation technique. World J. Cardiol..

[9] Colarossi G., Migliorini F., Becker M., Arias J.P., Autschbach R., Moza A., Aljalloud A. (2023). Conventional Prostheses versus Sutureless Perceval for Aortic Valve Replacement: A Meta-Analysis. Ann. Thorac. Cardiovasc. Surg..

[10] Hermanns H., Alberts T., Preckel B., Strypet M., Eberl S. (2023). Perioperative Complications in Infective Endocarditis. J. Clin. Med..

[11] Pizzino F., Paradossi U., Trimarchi G., Benedetti G., Marchi F., Chiappino S., Conti M., Di Bella G., Murzi M., Di Sibio S. (2024). Clinical Features and Patient Outcomes in Infective Endocarditis with Surgical Indication: A Single-Centre Experience. J. Cardiovasc. Dev. Dis..

[12] Asta L., Falco D., Benedetto U., Porreca A., Majri F., Angelini G.D., Sensi S., Di Giammarco G. (2024). Stroke after Cardiac Surgery: A Risk Factor Analysis of 580,117 Patients from UK National Adult Cardiac Surgical Audit Cohort. J. Pers. Med..

[13] Saito S., Sairenchi T., Hirota S., Niitsuma K., Yokoyama S., Kanno Y., Kanazawa Y., Tezuka M., Takei Y., Tsuchiya G. (2022). Prosthetic Valve Function after Aortic Valve Replacement for Severe Aortic Stenosis by Transcatheter Procedure versus Surgery. J. Cardiovasc. Dev. Dis..

[14] Hu J., Chen R., Liu S., Yu X., Zou J., Ding X. (2016). Global Incidence and Outcomes of Adult Patients With Acute Kidney Injury After Cardiac Surgery: A Systematic Review and Meta-Analysis. J. Cardiothorac. Vasc. Anesth..

[15] Çınar T., Hayıroğlu M.I., Tanık V.O., Aruğaslan E., Keskin M., Uluganyan M., Öz A., Çağdaş M., Alper A.T. (2018). The predictive value of the CHA2DS2-VASc score in patients with mechanical mitral valve thrombosis. J. Thromb. Thrombolysis.

[16] Agrawal A., Arockiam A.D., Jamil Y., El Dahdah J., Honnekeri B., El Helou M.C., Kassab J., Wang T.K.M. (2023). Contemporary risk models for infective endocarditis surgery: A narrative review. Ther. Adv. Cardiovasc. Dis..

[17] Di Eusanio M., Berretta P. (2020). The sutureless and rapid-deployment aortic valve replacement international registry: Lessons learned from more than 4500 patients. Ann. Cardiothorac. Surg..

